# What happens for informal caregivers during transition to increased levels of care for the person with dementia? A systematic review protocol

**DOI:** 10.1186/s13643-018-0755-0

**Published:** 2018-06-26

**Authors:** Marianne Cranwell, Anna Gavine, Linda McSwiggan, Timothy B. Kelly

**Affiliations:** 1 School of Education and Social Work, University of Dundee, Room 2.34 Carnelly Building, Dundee, DD1 4HN UK; 2 School of Nursing and Health Sciences, University of Dundee, Dundee, DD1 4HN UK

**Keywords:** Caregiver, Dementia, Transition, Placement, Institutionalisation

## Abstract

**Background:**

Dementia is a globally prevalent disease that requires ongoing and increasing levels of care, often provided in the first instance by informal caregivers. Supporting transitions in informal caregiving in dementia is a pertinent issue for caregivers, care providers and governments. There is no existing systematic review that seeks to identify and map the body of literature regarding the review question: ‘What happens for informal caregivers during transition to increased levels of care for the person with dementia?’

**Methods/design:**

ASSIA, CINAHL+, MEDLINE, PsycINFO, SCIE, Social Service Abstracts and Web of Science will be systematically searched. Specialist dementia research libraries will be contacted. Reviews identified as relevant during the search process, their reference lists, and reference lists of accepted papers will be hand-searched. Qualitative, quantitative and mixed methods studies that seek to represent the experiences of, or examine the impact upon, informal caregivers during transition to increased formal care for the person with dementia will be eligible for inclusion. Synthesis will be segregated into qualitative and quantitative papers. Findings will be summarised, and the review will be prepared for publication.

**Discussion:**

The review will seek to identify potentially vulnerable groups in need of support and as such, inform the practice of those offering support. It will also inform future research by highlighting areas in which current literature is insubstantial.

**Systematic review registration:**

PROSPERO CRD42017067248

**Electronic supplementary material:**

The online version of this article (10.1186/s13643-018-0755-0) contains supplementary material, which is available to authorized users.

## Background

Alzheimer’s Disease International’s World Alzheimer Report (2015) [1] estimated that in 2015, there were 46.8 million people living with dementia (PwD). Globally, this figure is predicted to increase to 131.5 million by 2050. The total figure for the UK in 2015 was estimated at 850,000 [2]. Recognition of the prevalence and projected increase of dementia diagnoses has so far led to approximately 29 national dementia plans, and the development of the World Health Organization’s Draft Global Plan on Public Health Response to Dementia 2017–2025.

In 2015, Carers UK [3] estimated the number of informal, or unpaid, caregivers in the UK to be 6.5 million, with the value of their caring input calculated in 2016 as £56.9 billion [4]. Informal care for PwD represented approximately 10% of this overall estimate of numbers of carers, with the value of the work contribution estimated at £11.6 billion [2]. However, identifying carers as carers is recognised as difficult, partly because carers themselves may not identify as ‘carers’ and partly because carers may not be known to services. This means that these national figures, and the values attributed to carers’ contributions, may be significantly underestimated.

Whilst anecdotally many caregivers report positive experiences of caring, the literature highlights that being an informal caregiver for PwD can pose risks to the physical and mental wellbeing of caregivers, through increased anxiety, burden, depression and physical symptoms [5–8]. When informal caregivers receive insufficient support or treatment for the negative aspects of caregiving, there is also a risk to the PwD of early institutionalisation [9–11].

As dementia is a progressive disease, the care needs of a PwD will inevitably increase. This means that informal caregivers will experience transitions when entering, during and ending their caregiving career. The review will focus on the period of time in which formal care is beginning, or increasing for the PwD, and how that is experienced by the informal caregiver. The prevalence of dementia, along with the related demands that this puts on informal caregivers, points to the necessity of mapping and understanding existing literature around transitions for informal caregivers of PwD. This review will lay the foundations required to develop strategies that support caregivers to have positive, healthy transitions.

The overarching review question is ‘What happens for informal caregivers during transition to increased levels of care for the PwD?’ There are two sub-questions: ‘How are transitions to higher levels of care for PwD experienced by their informal caregivers?’ and ‘How do transitions to higher levels of care for PwD impact on their informal caregivers?’

### Preliminary searches

During preliminary scoping searches, Afram et al.’s [12] qualitative review of the needs of informal caregivers when the PwD moves to institutional care was identified. This is strongly connected to the topic of the current review, in which it is intended that all possible transitions to increased care will be included rather than only those in which PwD move to institutional care.

A search of the International Prospective Register of Systematic Reviews (PROSPERO) has been undertaken to support the review’s originality. Two review protocols that related to the review topic but were not directly equivalent were found. Both protocols have a narrower focus than the current review: One sought to address the stability of home-based care for PwD [13], and one focused on interventions for PwD and their caregivers during transition from home care to nursing home [14].

### Definitions and terminology

Variation in terminology may be problematic, and therefore, to enhance clarity within this review, it is important to define key terms; these definitions are not exhaustive but will act as guidance during the review process.

For the purposes of this review, ‘caregivers’ will refer to people who support PwD in daily tasks on a regular basis (this may include anything from providing company, support with medication, personal care or maintaining personal safety). Within the wider literature, the distinction between formal and informal caregivers is often imprecise. Within this review, ‘informal caregiver’ will be used to refer to people who provide regular, uncontracted support; ‘formal caregiver’ will refer to a person who is paid to provide a specified service.

The phrase ‘increased level of care’ denotes circumstances where formal care commences either in addition to that which is provided by the informal caregiver or replaces that of an informal caregiver. This may include the introduction of formal care in the home, commencement of regular visits to a day centre or admission to a permanent residential facility or hospital.

## Methods

### Objectives

The objectives of the review are, first, to systematically identify and describe the current body of literature on the experiences of caregivers of PwD during transition to higher level of care, or the impact of that transition on caregivers, and secondly, to synthesise current evidence to map any apparent commonalities and disparities in emergent themes.

### Review method

This systematic review protocol was developed using the PRISMA-P checklist [15, 16] and will be registered on PROSPERO. The PRISMA checklist and flow chart [17] will be used throughout the review. The review will follow the EPPI-Centre approach as detailed in Gough et al. [18]. Following the standard systematic review format, the review will focus on the inclusion of both qualitative and quantitative literature, and both the appraisal and relevance of accepted articles.

### Eligibility criteria

To meet the aims of a broad exploratory systematic review, studies published in English, in peer reviewed journal, during any publication period will be accepted. Grey literature that meets eligibility criteria with the exception of peer review will be accepted.

Participants in studies will be informal caregivers of PwD. The person with dementia must have a formal diagnosis of dementia. Formal recognition of participants’ role as a caregiver (by an institution or organisation such as a caregiving charity) is not necessary. Where more than one group of participants are included in a study, data relating to informal caregivers must distinct; studies that do not distinguish clearly between data from informal caregivers and other participants (for example, people with dementia or professionals) will be excluded.

The topic of studies should be a period of transition in which the level of care provided to the PwD is being increased in any context (community or residential) with the intention of it being a permanent change in care arrangements. Studies that focus on transition to lower levels of support (for example, discharge from hospital) or temporary transitions (for example, crisis respite, trial periods) will be excluded. Studies in which interventions that support transitions are being tested will also be excluded. Where papers explore both permanent and temporary transitions in care, only papers with distinct data relating to permanent transitions will be included in the review. Studies must seek to either represent the experience of informal caregivers or to measure the impact of transition on informal caregivers. Where impact is measured, outcomes must be clearly stated and related to informal caregivers (for example, measurement scales of level of depression, quality of life or burden).

Both qualitative and quantitative designs will be included. More specifically, we will include cohort studies (retrospective and prospective), case-control studies and cross-sectional studies that measure quantitative data. Only cohort studies and case-control studies will be pooled in the meta-analysis. We will include qualitative studies of any design, provided that the methods and results of the study are clearly presented, and all other criteria are met. The review’s purpose of describing experience denotes that intervention studies (e.g. randomised controlled trials, quasi-experimental studies) will be excluded.

### Information sources

Databases to be searched will include ASSIA (via ProQuest), Cumulative Index to Nursing and Allied Health Literature (CINAHL+) (via EBSCOhost), MEDLINE (via OVID, all papers 1946 onwards), PsycINFO (via EBSCOhost), Social Care Institute for Excellence (SCIE), Social Services Abstracts (via ProQuest) and Web of Science. Web of Science Core Collection includes Science Citation Index Expanded (1900–present), Social Sciences Citation Index (1900–present), Arts & Humanities Citation index (1975–present), Conference Proceeding Index—Science (1900–present), Conference Proceedings Citation Index—Social Science and Humanities (1990–present), Book Citation Index—Science (2005–present), Book Citation Index—Social Sciences & Humanities (2005–present) and Emerging Sources Citation Index (2015–present). Grey literature will be obtained through direct contact with specialist organisations and a general Google search limited to the first 100 results.

Specialist resources including Alzheimer Scotland Dementia Research Centre, Alzheimer’s Research UK, Alzheimer’s Society Dementia Catalogue, Carer’s Trust, Carers UK, Dementia Services Development Centre Library and UK Dementia Research Institute will be contacted to identify grey literature.

### Search strategy

The databases listed above will undergo comprehensive individualised searches, using index terms and free text relating to dementia, caregiving and transitions (see Additional file 1).

Relevant literature reviews will be identified during the search process, and all articles recommended by specialist resources will be accessed. Reference lists of these articles along with those of studies included in the review will be hand-searched. Search terms will be developed using a combination of free-text terms with Boolean limiters, truncation and index term searching.

### Study records

#### Data management

A manual search log will be kept of databases searched and search terms used. Numerical search results will be reported in the PRISMA [17] flow diagram. Zotero reference manager will be used to record citations, abstracts and full texts and to identify duplicates.

#### Selection process

Two reviewers will screen titles and abstracts independently for potential eligibility for inclusion in the review. Full papers will be obtained if eligibility is unclear or established. Authors will be contacted for clarification or additional data where necessary, and a third reviewer will be available to resolve any disagreement.

#### Quality assessment and risk of bias

Papers will be assessed using Gough’s 2007 Weight of Evidence Framework [19], the first dimension of which (quality assessment of individual studies) will use the following tools: Quality of qualitative and mixed methods studies will be assessed using a checklist of narrative prompts based on those proposed by Walsh and Downe [20] (see Additional file 2). The checklist will be minimally modified to integrate the explicit assessment of validity proposed in Sandelowski and Barroso [21]. Quantitative critical appraisal will be assessed using the Newcastle-Ottawa scale [22] for cohort and case-control studies, or the Appraisal Tool for Cross-Sectional Studies (AXIS) [23]. Rather than a separate risk of bias assessment for qualitative papers, reflexivity, trustworthiness and validity will be an integrated element of quality assessment.

#### Meta biases

If sufficient studies reporting quantitative data for each outcome are available (defined as 10 or more), we will generate funnel plots for each outcome to assess for publication bias. The funnel plots will be inspected visually, and the Egger test [24] will be used to test for funnel plot asymmetry.

We will explore selective outcome reporting by identifying any existing study protocols and comparing the outcomes reported in the protocol with the outcomes reported in the paper. A reflexive account will be developed to address risk of bias within the review.

#### Data extraction

Quantitative and qualitative data will be extracted using different data extraction tables. Mixed methods data will be separated during data extraction for synthesis. Standardised tables will be used for quantitative data and will include the following headings: research question, study location, setting and topic, sample size, study duration and design, and results/findings. For qualitative data, tables will be developed based on the guidance of Evans and Pearson [25] and Sandelowski et al. [26]. Data extraction tables will be piloted prior to proceeding.

#### Outcomes

Two strands of outcomes and phenomena of interest will be equally prioritised: experience and impact. For papers focused on the experience of the transition, quotes, phrases and findings may be described in terms of belief, experience, feelings, knowledge, perception or understanding. We anticipate that these are likely to be qualitative data, which pertain to the phenomenon of interest.

For the purposes of this review, impact measurement will be considered separately in terms of quantitative and qualitative data. Quantitative data will be used to examine the impact that the care transition has on specific health outcomes of informal caregivers, namely quality of life, depression and/or anxiety, wellbeing and burden. Qualitative data will be used to identify examples of how the transition has affected the informal caregivers lives (e.g. change in job, relationship effects).

#### Data synthesis

Synthesis of findings will be undertaken using a segregated approach [27, 28]. Data will be grouped into qualitative or quantitative, and thematic synthesis will be performed on each group to form the primary synthesis.

Quantitative data obtained from cohort or case-control studies will be entered into Review Manager 5 software [29] and synthesised using different meta-analyses for the following outcome measures: quality of life, depression and/or anxiety, wellbeing and burden. We anticipate some heterogeneity in terms of populations studied, and a random-effects model will therefore be used to combine the data. For dichotomous data, we will present results as risk ratios with 95% confidence intervals. For continuous data, we will present results as standardised mean difference with 95% mean difference. In the case of missing data, we will contact study authors to attempt to obtain the data. If we are still unable to obtain the data, the study will not be included in the meta-analysis and will be described narratively instead. In the case that there is insufficient studies to perform a meta-analysis, where data is presented (or obtained from study authors), we will calculate a common effect size to allow for comparability between studies and present this in a narrative synthesis. Dichotomous data will be presented as risk ratio (and 95% confidence interval), and continuous data will be presented as a mean difference (and 95% confidence interval).

Statistical heterogeneity will be assessed in each meta-analyses using *τ*^2^, *I*^2^ and χ^2^ statistics. If substantial heterogeneity is identified (defined as *I*^2^ > 50%), we will explore it using the following sub-group analyses: gender of informal caregiver; relationship of informal giver to PwD (i.e. child, spouse, sibling, friend); context (i.e. community versus residential); nature of care provider (i.e. providing company, support with medication, personal care or maintaining personal safety); duration of informal caregiving (i.e. more or less than 6 months); and study design (i.e. cohort study versus case control).

Any quantitative data obtained from cross-sectional studies will not be included in the meta-analysis and will be presented narratively.

Secondary synthesis will incorporate all papers and seek to bring the findings from primary syntheses together. Thematic synthesis will be performed on qualitative data [18, 30], following the process of coding text, developing descriptive themes and then generating analytical themes. This process will be undertaken by one researcher, checked by a second and any disparities discussed with a third. A grid that enables the researcher to visualise the relative contribution of each study will be compiled to aid scrutiny of the robustness of the synthesis [18].

## Discussion

The review will provide a comprehensive overview of the current body of literature regarding the experiences of, and impact upon, informal caregivers during periods of transition to increased levels of care for the PwD. A limitation of the review will be its restriction to English-only sources; this is due to the time and resources available to the reviewers. The findings of the review will identify those who may be at increased risk of harm during the transition period and the nature of that harm. This will aid development of practice for organisations involved in the transition period and help to prioritise areas that require additional support. The review will also act as a basis for further research.

## Additional files

Additional file 1: Search strategies. (DOCX 19 kb)

Additional file 2: Quality assessment prompts for qualitative and mixed methods papers. (DOCX 19 kb)

## Abbreviations

ASSIA: Applied Social Sciences Index and Abstracts
AXIS: Appraisal Tool for Cross-Sectional Studies
CINAHL: Cumulative Index of Nursing and Allied Health Literature
EPPI-Centre: Evidence for Policy and Practice Information and Co-ordinating Centre
PRISMA: Preferred Reporting Items for Systematic Reviews and Meta-Analyses
PwD: Person with dementia
SCIE: Social Care Institute for Excellence
WHO: World Health Organization
