# Supplementary material for: What happens for informal caregivers during transition to increased levels of care for the person with dementia? A systematic review protocol
Source: Syst Rev. 2018 Jun 26;7:91. doi: 10.1186/s13643-018-0755-0 (PMC6020322; doi:10.1186/s13643-018-0755-0)
Supplement: Supplementary file 1 — Search strategies. (DOCX 19 kb) [file 13643_2018_755_MOESM1_ESM.docx]

**Additional file 1: Search strategies for all databases and draft email to specialist resources.**

**ASSIA**

((dementia OR Alzheimer*)

AND

(carer* OR caregiver*)

AND

(transition* OR institution* OR relinquish* OR adjust* OR acclimat* OR adapt*))

AND

(stype.exact("Scholarly Journals") AND PEER(yes))

On 27/07/17 = 3244

**CINAHL+**

((MH ‘dementia+’ OR ‘dementia’)

AND

(‘MH caregivers’ OR ‘caregiver*’ OR ‘carer*’ OR MH ‘home nursing’)

AND

(MH ‘acclimatization’ OR MH ‘life change events’ OR ‘MH ‘relocation’ OR MH ‘adaptation, psychological+’ OR MH ‘adaptation, physiological+’ OR MH ‘institutionalization+’ OR ‘acclimat*’ OR ‘adjust*’ OR ‘institution*’ OR ‘relinquish*’ OR ‘transition*’ OR ‘adapt*’)

On 26/06/17 =1783

**COCHRANE**

(Dementia OR alzheimer*)

AND

(carer* AND caregiver*)

AND

(transition* OR institution* OR relinquish* OR adjust* OR acclimat* OR adapt* OR home nursing OR life change events)

On 20/09/17 = 1141

**MEDLINE**

(‘exp dementia’ OR ‘dementia’)

AND

(‘exp home nursing’ OR ‘exp caregivers’ OR ‘social support’ OR ‘carer*’ OR caregiver*’)

AND

(‘exp adaptation, physiological’ OR ‘adapt*’ OR ‘exp adaptation, psychological’ OR ‘acclimatization’ OR ‘acclimat*’ OR ‘exp life change events’ OR ‘adjust*’ OR ‘exp social adjustment’ OR ‘exp emotional adjustment’ OR ‘relocation’ OR ‘relinquish*’ OR ‘institution*’ OR ‘transition*’)

On 18/07/17 = 2376

**PSYCINFO**

(DE ‘dementia’ exp OR DE ‘Alzheimer’s Disease’ OR ‘Alzheimer*’ OR ‘dementia’)

AND

(DE ‘caregivers’ OR ‘carer*’ OR ‘caregiver*’

AND

(DE ‘adjustment’ exp OR DE ‘home care’ OR ‘transition*’ OR ‘acclimat*’ OR ‘adapt*’ OR ‘adjust*’ OR ‘institution*’ OR ‘relocat*’ OR ‘relinquish*’)

On 30/08/17 = 2118

**SOCIAL CARE ONLINE**

(‘dementia*’ OR ‘Alzheimer*’)

AND

(‘caregiver*’ OR ‘carer*’)

AND

(‘transition*’ OR ‘institution*’ OR ‘relinquish*’ OR ‘adjust*’ OR ‘acclimat*’ OR ‘adapt*’)

On 29/08/17 = 185

**SOCIAL SERVICES ABSTRACTS**

(‘Dementia’ OR ‘Alzheimer*’)

AND

(‘carer* OR ‘caregiver*’)

AND

(‘transition*’ OR ‘institution*’ OR ‘relinquish*’ OR ‘adjust*’ OR ‘acclimat*’ OR ‘adapt*’

AND

(stype.exact ‘scholarly journal’s AND PEER (yes)

On 27/07/17 = 2675

**WEB OF SCIENCE**

(TS ‘dementia’ OR ‘alzheimer*’)

AND

(TS ‘carer*’ OR ‘caregiver*’)

AND

(TS ‘transition*’ OR ‘institution*’ OR ‘relinquish*’ OR ‘adjust*’ OR ‘acclimat*’ OR ‘adapt*’)

AND

Language: English AND Document type: Article or Review

On 12/09/17 = 2192

**Email sent to specialist resources 16/05/17 and 13/09/17:**

Dear Sir/Madam,

As part of my PhD study on experiences of transitions for informal caregivers of people living with dementia, I am working on a systematic literature review. The review question is ‘What happens for informal caregivers, during transition to increased levels of care for the person with dementia?’

The review seeks explore and map existing literature. It will include any methodology provided the focus of the paper is on the experience of informal caregivers (such as family, partner) or the impact of the experience on the caregiver. Increased levels of care will refer to the commencement of home care, regular attendance at day centres, permanent admission to hospital, institutionalisation, moving into sheltered housing and so on.

I wanted to contact you to ask if you may know of any papers that may meet the criteria for inclusion in this review, or are aware of any specialist resources that I might not yet be aware of?

Any input would be very gratefully received.

Thank you for your time,

Marianne Cranwell
