# Supplementary material for: What happens for informal caregivers during transition to increased levels of care for the person with dementia? A systematic review protocol
Source: Syst Rev. 2018 Jun 26;7:91. doi: 10.1186/s13643-018-0755-0 (PMC6020322; doi:10.1186/s13643-018-0755-0)
Supplement: Supplementary file 2 — Quality assessment prompts for qualitative and mixed methods papers. (DOCX 19 kb) [file 13643_2018_755_MOESM2_ESM.docx]

**Additional file 2: Quality Assessment Prompts for Qualitative and Mixed Method Papers** [1, 2]

| Part 1: Walsh and Downe [1] |  |  | |
| --- | --- | --- | --- |
| **Stages** | **Essential Criteria** | **Specific Prompts** | |
| Scope and purpose | Clear statement of, and rationale for, research question/aims/purposes | - Clarity of focus demonstrated - Explicit purpose given, such as descriptive/explanatory intent, theory building, hypothesis testing - Link between research and existing knowledge demonstrated | |
|  | Study thoroughly contextualised by existing literature | - Evidence of systematic approach to literature review, location of literature to contextualise the findings, or both | |
| Design | Method/design apparent, and consistent with research intent | - Rationale given for use of qualitative design - Discussion of epistemological/ontological grounding - Rationale explored for specific qualitative method (e.g. ethnography, grounded theory, phenomenology) - Discussion of why particular method chosen is most appropriate/sensitive/relevant for research question/.aims - Setting appropriate | |
|  | Data collection strategy apparent and appropriate | - Were data collection methods appropriate for type of data required and for specific qualitative method? - Were they likely to capture the complexity/diversity of experience and illuminate context in sufficient detail? - Was triangulation of data sources used if appropriate? | |
| Sampling strategy | Sample and sampling method appropriate | - Selection criteria detailed, and description of how sampling was undertaken - Justification for sampling strategy given - Thickness of description likely to be achieved from sampling - Any disparity between planned and actual sample explained | |
| Analysis | Analytic approach appropriate | - Approach made explicit (e.g. thematic distillation, constant comparative method, grounded theory) - Was it appropriate for the qualitative method chosen? - Was data managed by software package or by hand, and why? - Discussion of how coding systems/conceptual frameworks evolved - How was context of data retained during analysis - Evidence that the subjective meanings of participants were portrayed - Evidence of more than one researcher involved in stages if appropriate to epistemological/theoretical stance - Did research participants have any involvement in analysis (e.g. member checking) - Evidence provided that data reached saturation or discussion/rationale if it did not - Evidence that deviant data was sought, or discussion/rationale if it was not | |
| Interpretation | Context described and taken account of in interpretation | - Description of social/physical and interpersonal contexts of data collection - Evidence that researcher spent time ‘dwelling with the data’, interrogating it for competing/alternative explanations of phenomena | |
|  | Clear audit trail given | - Sufficient discussion of research processes such that others can follow ‘decision trail’ | |
|  | Data used to support interpretation | - Extensive use of field note entries/verbatim interview quotes in discussion of findings - Clear exposition of how interpretation led to conclusions | |
| Reflexivity | Researcher reflexivity demonstrated | - Discussion of relationship between researcher and participants during fieldwork - Demonstration of researcher’s influence on stages of research process - Evidence of self-awareness/insight - Documentation of effects of the research on researcher - Evidence of how problems/complications met were dealt with | |
| Ethical dimensions | Demonstration of sensitivity to ethical concerns | - Ethical committee approval granted - Clear commitment to integrity, honesty, transparency, equality and mutual respect in relationships with participants - Evidence of fair dealing with all research participants - Recording of dilemmas met and how resolved in relation to ethical issues - Documentation of how autonomy, consent, confidentiality, anonymity were managed | |
| Relevance and transferability | Relevance and transferability evident | - Sufficient evidence for typicality specificity to be assessed - Analysis interwoven with existing theories and other relevant explanatory literature drawn from similar settings and studies - Discussion of how explanatory propositions/emergent theory may fit other contexts - Limitation/weaknesses of study clearly outlined - Clearly resonates with other knowledge and experience - Results/conclusions obviously supported by evidence - Interpretation plausible and ‘makes sense’ - Provides new insights and increases understanding - Significance for current policy and practice outlines - Assessment of value/empowerment for participants - Outlines further directions for investigation - Comment on whether aims/purposes of research were achieved | |
| Part 2: Sandelowski & Barosso [2] | | | |
| **Concept** | **Appraisal Parameters** | **Presence (yes/no)** | **Relevance (yes/no)** |
| Validity  Look for discussions of techniques specifically intended to unsure that the study is scientifically and/or ethnographically valid or ‘good’.  Included is information about the strength and limitations of a study, of specific topics such a reflexivity, reliability, rigor, credibility and plausibility, and of specific procedures such a member validation and peer review.   Information about validity may be explicitly stated, or implied in discussions of sampling, the sample, data collection and analysis, and in the presentation of the findings.  Researchers may emphasize, although not identify as such, different kinds of ‘validities’ in their study: e.g. descriptive, interpretive, theoretical, and pragmatic validity. | 1. Researchers show an awareness of their influence on the study and its participants |  |  |
|  | 2. The distinctive limitations of the study are summarized: e.g. theoretical sampling could not be fully conducted in a grounded theory study. This is in contrast to summarizing and/or apologizing for the so-called limitations of qualitative research. |  |  |
|  | 3. Techniques for valuation are used that fit the purpose, method, sample, data, and findings, as opposed to using techniques that do not fit as when reliability coding to ascertain consistency in interview data is used in a study emphasizing the revisionist nature of narratives. |  |  |
|  | 4. Techniques used are tailored to the reported study, as opposed to presentations of textbook or rote descriptions of validation techniques with no application shown to the study reported. |  |  |
|  | 5. Techniques for validation are accurately rendered, as opposed to misrepresented as when descriptive validity is confused with interpretive validity, and triangulation for convergent validity is confused with using different data sources for completeness. |  |  |
|  | 6. Techniques for validation are correctly used, as opposed to incorrectly used as when cases are kept in or dropped from consideration because they conform or do not conform to other cases. |  |  |

References:

[1] Walsh D, Downe S. Appraising the quality of qualitative research. Midwifery. 2006; 22(2):108–19.

[2] Sandelowski M, Barroso J. Reading qualitative studies. International Journal of Qualitative Methods. 2002; 1(1):74–108.
